# Use of apertures in single‐energy pristine Bragg peak FLASH radiotherapy

**DOI:** 10.1002/acm2.70593

**Published:** 2026-04-28

**Authors:** Yangguang Ma, Balaji Selvaraj, Xingyi Zhao, Chingyun Cheng, Chin‐Cheng Chen, Longfei Diao, Yufei Wang, Zhengda Wang, Yuntong Pei, Lele Liu, Xueqing Yan, Benjamin Durkee, Charles B. Simone, Haibo Lin, Xuanqin Mou, Minglei Kang

**Affiliations:** ^1^ Department of Radiation Oncology The First Affiliated Hospital of Zhengzhou University Zhengzhou Henan China; ^2^ New York Proton Center New York New York USA; ^3^ Department of Radiation Medicine University of Wisconsin School of Medicine and Public Health Madison Wisconsin USA; ^4^ Department of Radiation Oncology St. Jude Children's Research Hospital Memphis Tennessee USA; ^5^ School of Physics Peking University Beijing China; ^6^ School of Software Engineering Xi'an Jiaotong University Xi'an Shaanxi China; ^7^ School of Information and Communications Engineering, Faculty of Electronic and Information Engineering Xi'an Jiaotong University Xi'an Shaanxi China

**Keywords:** aperture, Bragg peak FLASH radiotherapy, penumbra reduction, proton pencil beam scanning

## Abstract

**Background:**

Proton single‐energy Bragg peak (SEBP) FLASH delivery can achieve dosimetric distributions equivalent to conventional intensity‐modulated proton therapy (IMPT). However, range‐pulling and field compensator devices enlarge the proton pencil beam spot size, increasing lateral penumbra and compromising dose conformality and high‐dose‐rate distribution. Apertures are employed to mitigate these effects, enhancing dose metrics while preserving ultra‐high dose rate performance.

**Purpose:**

To investigate the efficacy of apertures in sharpening lateral dose falloff and enhancing dose conformity in proton pencil beam scanning (PBS) Bragg peak (BP) FLASH radiotherapy (RT), addressing increased lateral dose spillage caused by universal range shifter (URS) and range compensator (RC) usage.

**Methods:**

PBS Single‐energy BP (SEBP) FLASH treatment plans with and without brass apertures were optimized using an in‐house planning system. Dose and dose rate characteristics were simulated using MCSquare. Penumbra reduction was assessed in a water phantom for 3 and 5 cm square fields under varying pullback (10 cm, 20 cm. 30 cm) and air gap (5 cm, 10 cm, 15 cm) conditions. Aperture effects were evaluated at the entrance, midpoint, and BP positions. The rGBM cancer plan was optimized using SEBP with various dose thresholds (0, 2, and 5 Gy) applied to analyze dose and ultra‐high dose rate (V_40Gy(RBE)/s_) performance.

**Results:**

Use of aperture significantly reduced lateral penumbra across all spatial positions. The degree of penumbra reduction increased significantly with the increased pullback, and the reduction effect at the BP was generally comparable to that at the entrance. The 5 cm field generally showed greater penumbra reduction than the 3 cm field. In the rGBM case, apertures improved gross tumor volume (GTV) dose conformity and reduced organ‐at‐risk (OAR) exposure but decreased ultra‐high dose rate coverage for both GTV and OARs. The ultra‐high dose rate coverage of GTV was not affected by the dose threshold, while the ultra‐high dose rate coverage of the brain increased with the increasing dose threshold.

**Conclusion:**

Apertures effectively reduce lateral penumbra and dose spalliage to OARs, improving target dose conformity in range shifter‐based SEBP FLASH‐RT. They also can reduce ultra‐high dose‐rate exposure to critical OARs in the low‐dose region while maintaining the FLASH ratio in the high‐dose region.

## 1 INTRODUCTION

FLASH radiotherapy (FLASH‐RT), employing ultra‐high dose rates (> 40 Gy/s), demonstrates significant normal tissue sparing while maintaining tumor control efficacy comparable to conventional radiotherapy (RT).^1^, ^2^, ^3^, ^4^, ^5^ This promising effect has accelerated efforts to translate FLASH‐RT into clinical practice. While most studies utilize electron or proton beams, with fewer exploring X‐rays, each modality presents unique challenges for clinical applications.^6^, ^7^, ^8^, ^9^ Protons, however, offer distinct advantages as they deliver no exit dose to critical organs‐at‐risk (OARs) beyond the Bragg peak (BP). Moreover, clinical proton therapy systems often inherently provide ultra‐high dose‐rate beams, positioning proton FLASH‐RT as a promising avenue for clinical implementation without any significant beam upgrade. Consequently, increasing interest is in leveraging proton therapy for FLASH‐RT translational research ^10^, ^11^, ^12^, ^13^, ^14^.

Existing proton irradiation systems, utilizing double scattering, uniform (wobbling) scanning, or pencil beam scanning (PBS), achieve BP spread‐out through sequential multi‐energy layer irradiation, conformally encompassing the tumor. However, the FLASH‐RT requirement for ultra‐high dose rates is impeded by energy switching times, rendering current methods unsuitable. This limitation has spurred research into minimizing or eliminating energy switching, with monoenergetic beam approaches gaining prominence. Three monoenergetic proton beam techniques for FLASH conformal irradiation have been developed: Transmission Beam (TB) FLASH,^13^, ^15^, ^16^, ^17^ Single‐energy Bragg peak (SEBP) FLASH,^18^, ^19^, ^20^, ^21^, ^22^, ^23^ and Single‐energy spread‐out Bragg peak beam (SESOBP) FLASH.^24^, ^25^, ^26^, ^27^, ^28^ Among these techniques, SEBP offers advantages over TB by leveraging the BPs and high relative biological effectiveness (RBE) of protons, enhancing normal OAR protection by eliminating any exit doses. In contrast to the three‐dimensional compensators utilized in SESOBP (e.g., ridge filters), the field‐specific range compensators (RCs) and universal range shifters (URSs) employed in SEBP present fewer fabrication challenges, thus offering enhanced clinical scalability.

PBS can achieve highly conformal treatments with minimal peripheral tissue exposure, making it increasingly mainstream in proton therapy. However, its plan quality is heavily dependent on the characteristics of the lateral dose falloff, which are influenced by spot size, range‐shifter presence and nozzle‐to‐patient air gap. Smaller spot sizes enhance dose conformity, whereas larger sizes improve robustness at the cost of lateral normal tissue sparing.^29^ Range shifters, though essential, exacerbate scatter and broaden lateral penumbra. In SEBP FLASH, the absence of beam modification techniques like secondary beam focusing results in increased spot size. Moreover, in SEBP treatment planning, particularly for shallow‐seated targets, the highest proton energy of 250 MeV can travel up to 38 cm in water. To achieve distal range tracking for these targets, significant range pullback and compensation are required. However, the multiple Coulomb scattering (MCS) between the protons and the range pullback devices can substantially increase the spot size. Concurrently, implementing URS and RCs for range modulation substantially increases beam divergence. These factors collectively contribute to lateral penumbra blurring, adversely increasing the adjacent OAR doses.

The impact is especially critical in the treatment of complex tumors such as reirradiation glioblastoma multiforme (rGBM) and head‐neck cancers, in which the critical OARs are close by, and the preservation of critical organs is paramount. Although the FLASH effect may provide additional normal tissue sparing, preclinical studies suggest that this benefit is typically on the order of 20%–30% compared with conventional dose‐rate RT,^1^, ^2^, ^3^, ^30^, ^31^, ^32^, ^33^ This protective effect may be diminished when FLASH dose is delivered using a nonconformal treatment plan. The “as low as reasonably achievable” (ALARA) principle should still be adhered to in FLASH treatment planning, design, and delivery^34^. Therefore, penumbra control remains critically important in FLASH RT to maintain high quality of treatment planning. While minimizing the air gap between the URS and the skin was crucial for achieving a sharp lateral dose falloff, the effectiveness of this approach decreases at very shallow depths. A more promising strategy involves using an aperture downstream of the proton delivery path. This provides additional beam collimation, thereby reducing scattered radiation and penumbra width.^35^, ^36^, ^37^, ^38^, ^39^, ^40^, ^41^, ^42^, ^43^, ^44^, ^45^ Apertures, akin to collimation devices in photon RT, are field‐shaping devices made of materials like brass. While multiple studies have investigated aperture use in PBS proton delivery, quantifying and sharpening lateral penumbra, its efficacy in SEBP FLASH for penumbra restriction and dose confinement remains unclear. This study focuses on the aperture application in SEBP FLASH‐RT to evaluate the dosimetry and dose rate performance using this beam sharpening device for future applications.

This study utilized MCsquare, a Monte Carlo simulation tool,^46^ to evaluate the collimation effects of apertures in BP FLASH RT. Lateral beam profiles on a water phantom and dose and dose rate distributions for a representative re‐irradation gliblstoma (rGBM) patient were assessed to quantitatively evaluate the aperture performance in SEBP FLASH‐RT.

## 2 METHODS

### 2.1 BP FLASH‐RT

The SEBP Flash technique,^18^ employs unmodified “monoenergetic” proton beams generated by the accelerator through URS and beam‐specific RC to adjust the proton beam range, ensuring the BP precisely targets the distal end of the tumor. Subsequently, multi‐field cross‐irradiation achieves high‐dose regions and ultra‐high dose rates that conform to the target volume. This novel technique has gained widespread application in FLASH research across various treatment sites, including liver‐hypofractionated radiation therapy,^19^ head and neck cancers,^21^ thoracic malignancies,^18^, ^23^, ^47^ breast cancer,^22^ and prostate cancer.^20^, ^47^

The effectiveness of this method relies on three main components: RC and URS dimensioning determination, spot parameter optimization, and cumulative dose deposition calculation for field irradiation. URS and RCs work together to achieve distal tracking, pulling back the pristine BP using appropriate thickness to align them with the target volume distally. The URS consists of several polymethyl methacrylate (PMMA) plastic plates with variable thicknesses. The different combinations of range shifter plates can generate discrete range pullbacks to facilitate the BP distal tracking. The combinations of URS and contours of RCs were calculated by a ray tracing algorithm.^18^ Then, the inverse optimization process will optimize the spot map to generate sparse spot distribution and maintain a minimum MU/spot constraint to balance both dosimetry and dose rate characteristics for FLAH delivery.^48^

### 2.2 Implement aperture in BP FLASH treatment planning

**FIGURE 1:**
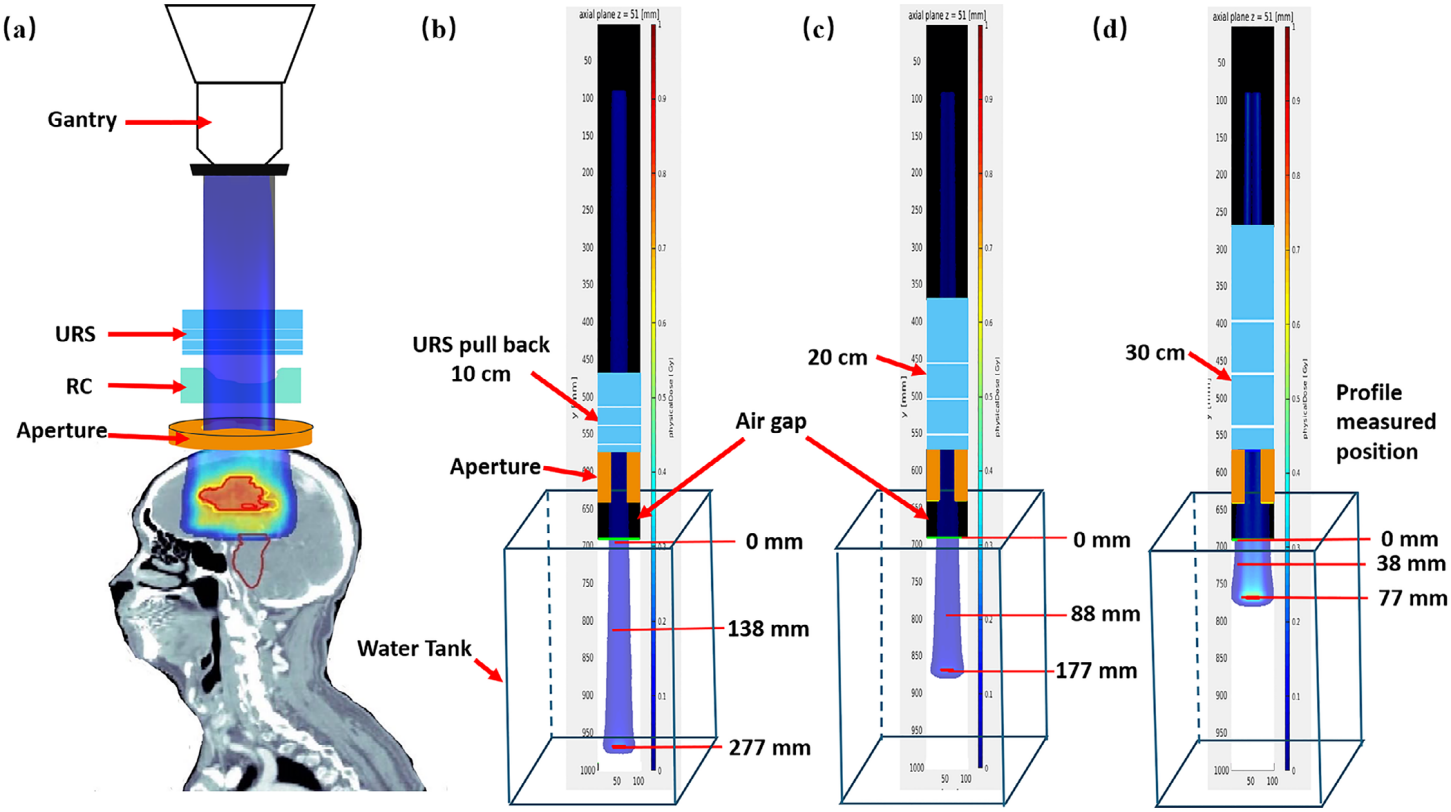
SEBP FLASH‐RT with brass aperture: (a) shows the SEBP technique using URS, RC, and brass aperture for a brain case treatment planning. (b) to (d) shows the penumbra changes in water phantom using different range pullbacks of 10, 20, and 30 cm under a 5 cm air gap between aperture and water phantom surface. The sky blue material in the beam path represent the URS marked with Bragg peak pull back by 10, 20, and 30 cm, respectively. All the black background represented the air.

A static field‐specific brass aperture was incorporated into the SEBP FLASH‐RT method to achieve sharp lateral dose falloff and highly conformal dose distribution. A 5 cm thick aperture is positioned downstream to minimize penumbra (Figure 1). The aperture margin, defined as the expansion from the target's beam's‐eye‐view (BEV) edge to the aperture's inner edge,^49^ is set to 5 mm. Penumbral width is defined as the distance between 80% and 20% isodose points on the beam edge profile.

An in‐house Treatment Planning System (TPS) was developed based on matRad.^50^ This TPS integrates MCsquare, a Monte Carlo toolkit, for SEBP treatment planning. The brass aperture was modeled on the beamline and used in treatment planning optimization and dose calculations. In this study, the ProBeam proton system's highest energy (250 MeV) was commissioned into MCsquare. One MU contains about 6.09 × 10^6^ protons. A single Gaussian was used, and the isocenter spot sigma for 250 MeV was 3.5 mm with a beam divergence of 3 mrad. The calculation grid size used was 2 mm, with a sufficient number of protons to maintain an accuracy level of 1%.

The dose rate distribution following PBS irradiation was assessed using the Average Dose Rate (ADR) paradigm.^51^ FLASH delivery is usually completed in less than 100 milliseconds, whereas beam switching time (i.e., gantry rotation time between different angles), which ranges from a few seconds to tens of seconds, was not included in the dose‐rate calculations. A crucial factor in FLASH beam delivery is the minimum spot time (MST), which directly influences the minimum MU applied in treatment planning for the ProBeam system.^19^, ^52^ Our BP plans were designed with a minimum MU of 400 and a MST of 0.5 ms.

### 2.3 SEBP FLASH‐RT uses aperture in water phantom

Proton SEBP FLASH beams were generated with and without apertures, employing air gaps of 5, 10, and 15 cm, pullbacks of 10, 20, and 30 cm, for two different field sizes of 3 × 3 cm^2^ and 5 × 5 cm^2^. Dose delivery to a water phantom was simulated using the MCsquare for these varied BP FLASH beam configurations. Then, lateral beam penumbra analysis was performed at the positions of the beam entrance, BP, and 50% range depth (Figure 1(b) to (d)). Based on that, the aperture's impact on the lateral penumbra was evaluated across all beam configurations.

### 2.4 Treatment planning validation for rGBM

An rGBM patient involving an intracranial tumor could potentially be a good candidate for proton SEBP FLASH‐RT, as the normal brain tissue has already received a significant dose from prior radiation therapy. A gross tumor volume (GTV) measuring 3.7 cm in diameter and 25.6 cc in volume was selected to demonstrate effectiveness. The treatment plan uses a hypofractionation regimen, prescribing 30 Gy(RBE) in 5 fractions. SEBP FLASH plans, both with and without apertures, were optimized. Three‐field irradiation was employed with beam angles of 270°/couch 0°, 90°/couch 270°, and 90°/couch 0°, with a 10 cm air gap. GTV dose coverage was normalized to ensure 100% of the volume received at least 95% of the prescribed dose for comparative analysis. A constant RBE value of 1.1 was applied for all dose optimization and calculation procedures.

Various dosimetric metrics were used to assess dose distribution, including maximum dose (D_max_) and mean dose (D_mean)_ for all OARs and the target. The conformity index (CI), based on radiation therapy oncology group (RTOG) guidelines,^53^ was calculated as the ratio of the total volume receiving the prescription dose to the total volume of the target, evaluating the conformity of high‐dose distribution. Dose distributions for all structures were detailed using a dose volume histogram (DVH). The V_12 Gy(RBE)_ of brain tissue has been reported to correlate with necrosis^54^, which was also evaluated here.

Considering the widely used minimum dose rate threshold of 40 Gy/s for the FLASH effect, V_40Gy(RBE)/s_ was utilized to indicate FLASH dose rate coverage, defined as the proportion of a region of interest (ROI) receiving dose at rates exceeding 40 Gy(RBE)/s. A dose rate volume histogram (DRVH) was employed to quantify dose rate statistics for target and OARs. A conservative per‐field approach was used to evaluate V_40Gy(RBE)/s_, with voxel‐wise dose rates calculated separately for each individual field. A voxel was included in the analysis only if the dose delivered by that field exceeded the specified threshold. Three minimum dose‐threshold scenarios were applied per voxel: 0 Gy(RBE), representing no dose threshold, 2 and 5 Gy(RBE). A detailed definition of V_40Gy(RBE)/s_ was described by Wei et al.^23^

## 3 RESULTS

### 3.1 SEBP FLASH‐RT uses aperture in water phantom

**FIGURE 2:**
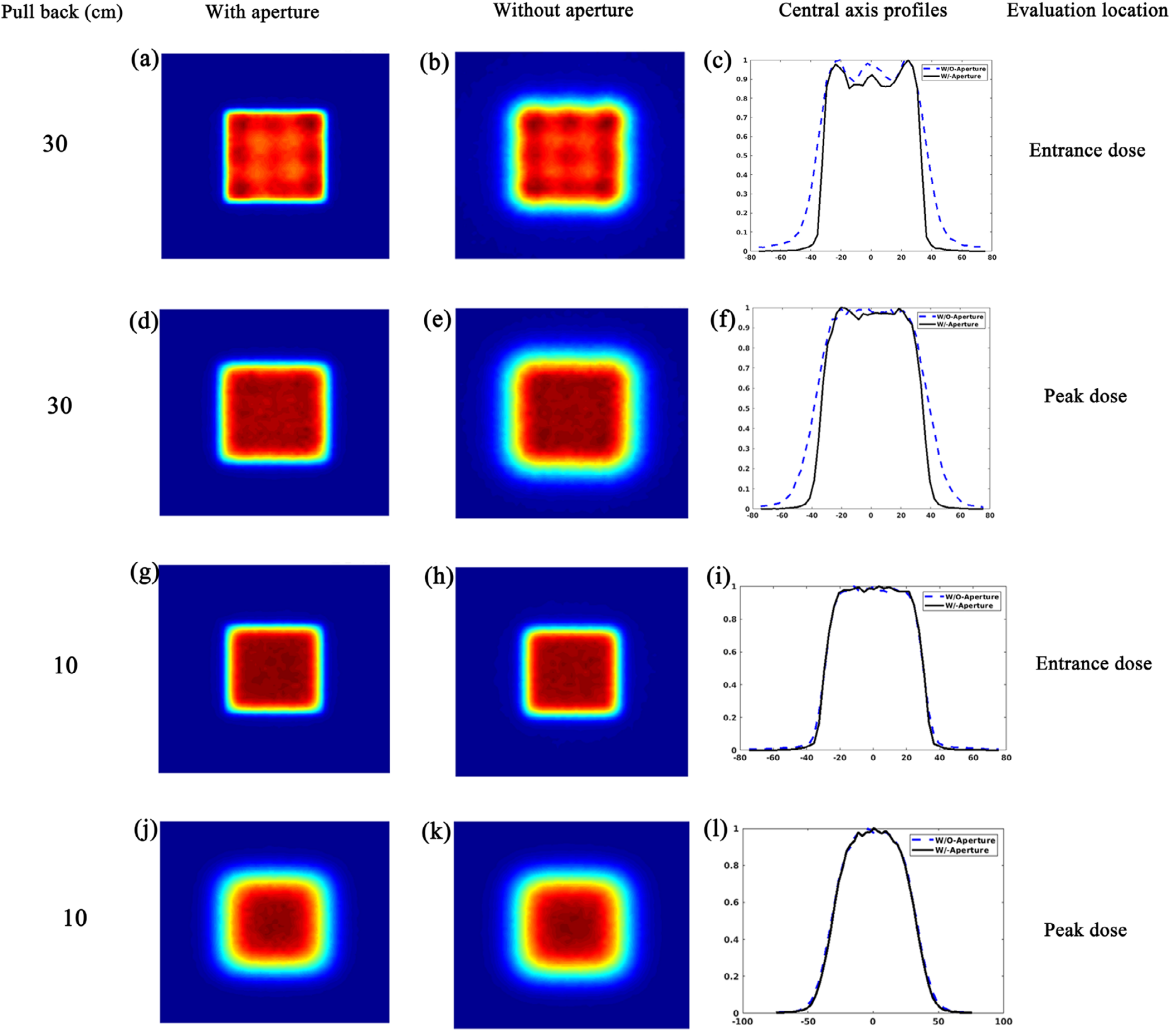
Comparative analysis of dose distributions and central axis profiles with and without brass aperture under varying conditions. The scenario uses a field size of 5 × 5 cm^2^, 5 mm spot spacing, 400 MU/spot, and 250 MeV proton under a 5 cm air gap.

**FIGURE 3:**
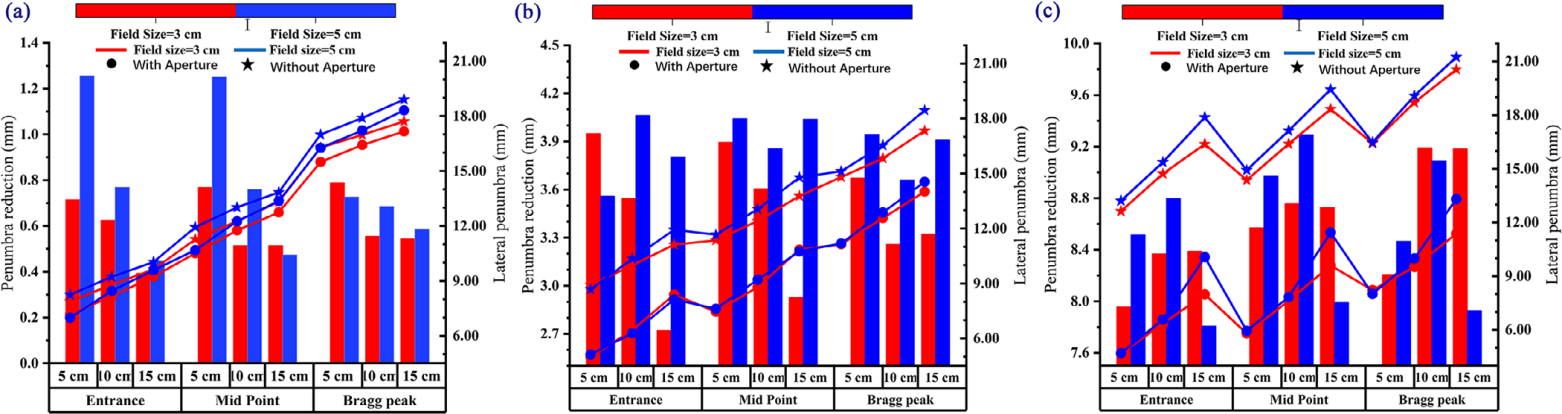
BP FLASH lateral penumbra size (point plot) and penumbra reduction values (bar) at pullbacks (a) 10 cm, (b) 20 cm, (c) 30 cm. Measurement locations: Entrance (phantom surface), Mid Point (mid‐range), Bragg Peak plane; Air gaps: 5, 10, 15 cm.

MCSquare Monte Carlo simulations of BP FLASH irradiation demonstrated a substantial lateral penumbra reduction with the implementation of an aperture (Figure 2). The penumbra reduction varied depending on field size, air gap, and pullback settings (Figure 3).

The penumbra characteristics of 3 × 3 cm^2^ and 5 × 5 cm^2^ field sizes with and without an aperture were investigated under various conditions. Without an aperture, the 3 × 3 cm^2^ fields generally showed smaller penumbras than the 5 × 5 cm^2^ fields. The penumbra values for the 3 × 3 cm^2^ and 5 × 5 cm^2^ field sizes, averaged across all designed scenarios (three depths and three air gaps), without an aperture, were as follows: 12.7 ± 3.4 mm vs. 13.3 ± 3.7 mm at a 10 cm pullback, 12.8 ± 2.6 mm vs. 13.4 ± 2.9 mm at 20 cm, and 16.5 ± 2.3 mm vs. 17.2 ± 2.4 mm at 30 cm. When using an aperture, penumbra values were reduced to 12.1 ± 3.4 mm vs. 12.6 ± 3.8 mm, 9.4 ± 2.8 mm vs. 9.5 ± 2.9 mm, and 7.9 ± 2.0 mm vs. 8.7 ± 2.6 mm for 3 × 3 cm^2^ and 5 × 5 cm^2^, respectively. This resulted in a more pronounced penumbra reduction effect for the 5 cm field size when using an aperture.

Figure 3 illustrates the lateral penumbra size with and without an aperture and the corresponding penumbra reduction at the phantom's surface, mid‐range, and BP plane under different geometric conditions. The line graph represents the penumbra size for each field, while the bar chart shows the reduction due to the aperture. As depicted, increasing the pullback distance consistently widened the penumbra without an aperture from 7.9∼18.9 mm (at 10 cm pullback) to 13.5∼21.3 mm (at 30 cm pullback). Conversely, with an aperture, the penumbra consistently narrowed as the pullback distance increased, from 7.2∼18.4 mm (at 10 cm pullback) to 4.7∼13.5 mm (at 30 cm pullback). This highlights the aperture's enhanced effectiveness in reducing the penumbra as the pullback increases, with reductions improving from 0.4∼1.3 (at 10 cm pullback) mm to 7.7∼9.4 mm (at 30 cm pullback). Moreover, at 30 cm pullback, the penumbra reduction effect for the two field sizes tended to converge under the same conditions.

The penumbra size increased with increased air gaps across all field sizes and conditions. For the 3 × 3 cm^2^ field, the aperture‐induced penumbra reduction decreased with increasing air gap at 10 and 20 cm pullbacks, with reductions ranging from 0.8 mm (5 cm air gap) to 0.4 mm (15 cm air gap) and 4.0 mm (5 cm air gap) to 2.7 mm (15 cm air gap), respectively. In contrast, at 30 cm pullback, the reduction increased from 7.7 mm (5 cm air gap) to 9.0 mm (15 cm air gap). For the 5 × 5 cm^2^ field, the penumbra reduction by aperture was weakened with increasing airgap at 10 cm pullback, with reductions between 1.3 mm (5 cm air gap) and 0.5 mm (15 cm air gap), while for 20 and 30 cm pullbacks, the effect was least (3.7 mm∼3.8 mm) or most pronounced (8.8 mm∼9.3 mm) at a 10 cm air gap.

### 3.2 Dose and dose rate performance in the rGBM

**FIGURE 4:**
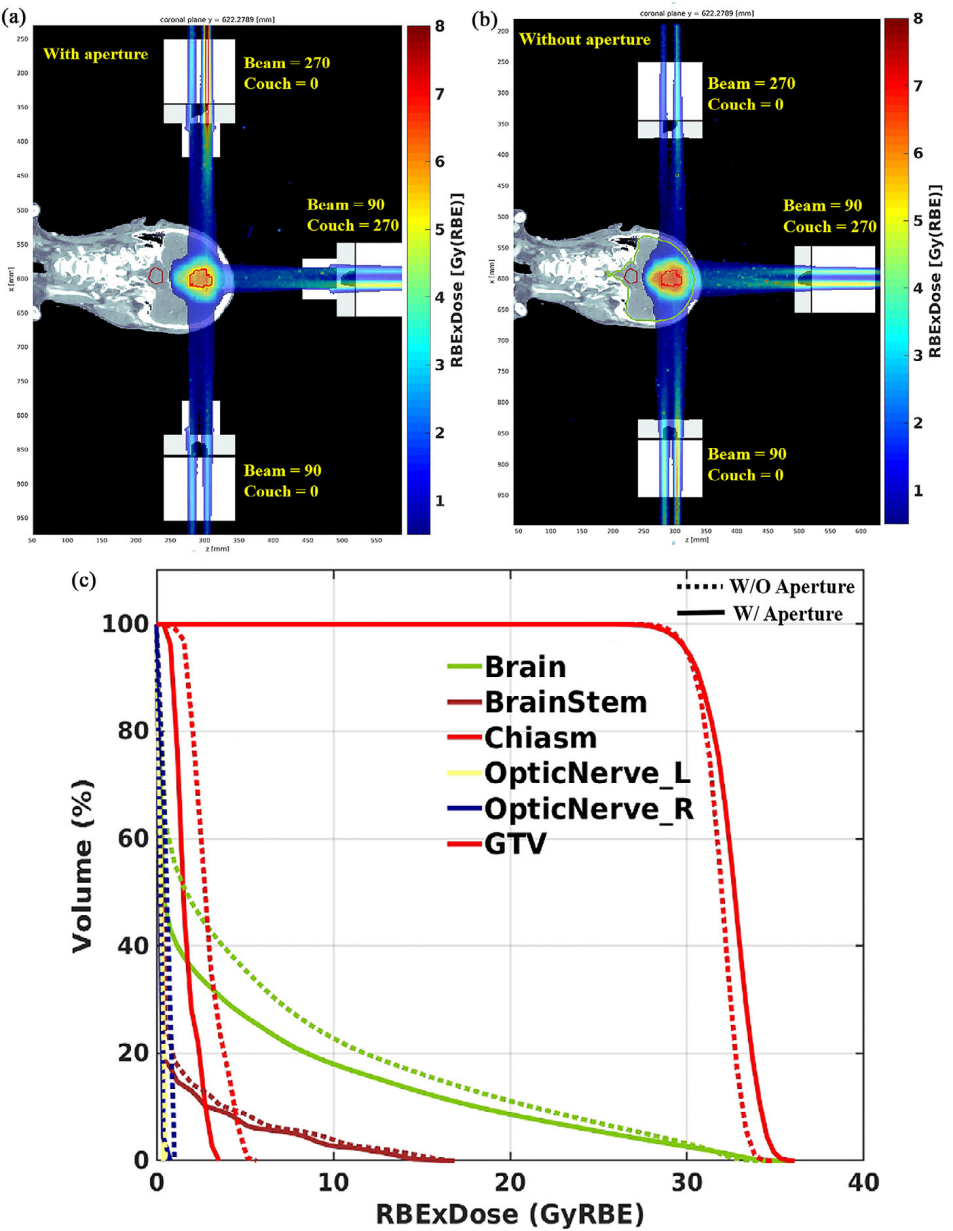
Dosimetric analysis of brain cancer treatment: (a,b) Planar dose distributions from BP FLASH irradiation with and without aperture, respectively; (c) DVHs for relevant structures.

Aperture‐integrated SEBP FLASH plans yield comparable target mean doses, [105.3% (31.59 Gy) vs. 105.3% (31.59 Gy(RBE))] and maximum doses [116.4% (34.92 Gy(RBE)) vs. 115.1% (34.53 Gy(RBE))], with improved conformity (CI: 1.3 vs. 1.4) compared to non‐aperture plans. They also demonstrate superior OAR sparing, with consistently lower DVHs. Aperture implementation reduced maximum doses in the brain stem, chiasm, and optic nerves from 54.7% (16.41 Gy(RBE)), 18.2% (5.46 Gy(RBE)), and 1.1% (0.33 Gy(RBE)) to 53.7% (16.11 Gy(RBE)), 10.9% (3.27 Gy(RBE)), and 0.6% (0.18 Gy(RBE)), respectively. The mean dose to the brain was reduced from 20.6% (6.18 Gy(RBE)) to 15.6% (4.68 Gy(RBE)). The V_12 Gy(RBE)_ was reduced from 19.7% to 15.8%. The 3D dose distributions (Figures 4a,b) and DVHs (Figure 4c) further corroborate the enhanced plan quality achieved with apertures.

**FIGURE 5:**
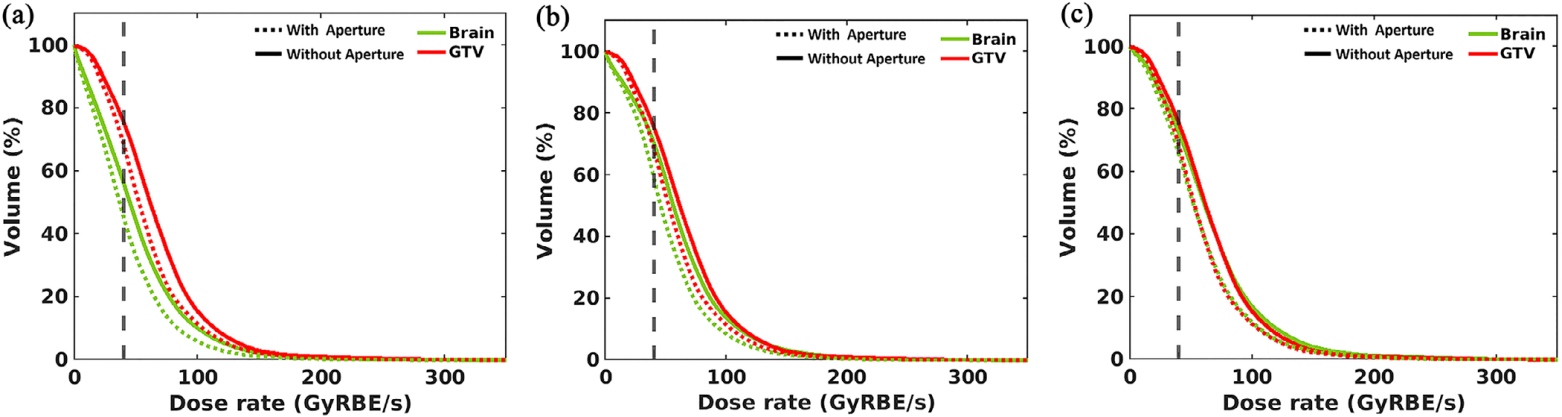
Dose rate comparison of BP FLASH planning for the rGBM with and without aperture at different minimum voxel dose thresholds: (a) 0 Gy(RBE), (b) 2 Gy(RBE), and (c) 5 Gy(RBE).

Across all three voxel dose thresholds (0, 2, and 5 Gy(RBE)) examined, aperture implementation can reduce the FLASH dose rate coverage for both the GTV and the brain (Figure 5). In the absence of an aperture, the V_40 Gy(RBE)/s_ for the GTV at thresholds of 0 Gy(RBE) (unrestricted), 2 Gy(RBE), and 5 Gy(RBE), were nearly identical, at 75.6%, 75.6%, and 75.6%, respectively. These values decreased to 68.3%, 68.3%, and 68.4% with the aperture. For the surrounding brain tissue, the V_40 Gy(RBE)/s_ values were 56.1%, 70.5%, and 72.5% without the aperture and decreased to 45.3%, 59.0%, and 65.7% with the aperture under the three dose thresholds. As the dose threshold increases to 5 Gy(RBE), the reduction in V_40 Gy(RBE)/s_ becomes minimal, and the V_40 Gy/s_ values for the brain and GTV converge. Thus, at the 5 Gy(RBE) dose threshold, the aperture did not significantly impact the FLASH ratio, as it effectively reduced low‐dose spillage.

## 4 DISCUSSION

This study introduces an aperture in the proton PBS SEBP FLASH‐RT to mitigate the large lateral penumbra. Using an in‐house TPS, we evaluated the aperture impact on lateral penumbra reduction in phantom and an rGBM hypofractionation treatment planning. Results reveal that BP FLASH with aperture significantly reduces lateral low‐dose spillage, improves target conformity, and enhances OAR protection. Positioned downstream of the URS and RCs as shown in Figure 1(a), the aperture filters scattered radiation from MCS between protons and the RCs and URS.

This study reveals that the lateral penumbra increases with depth regardless of aperture use, aligning with the existing literature. However, aperture‐induced penumbra reduction exhibits depth‐dependent variations (Figure 3), contrasting with conventional multiple‐energy PBS treatment, where reduction consistently increases with depth.^38^ The penumbra and its reduction introduced by the aperture increase with greater pullback distances across all field sizes. Penumbra also tends to increase with larger air gaps. However, the aperture's reduction effect varies with air gap changes, depending on pullback distance, field size, and evaluation position. At a 10 cm pullback, the aperture's impact decreases as the air gap increases for all field sizes. With a 20 cm pullback, the reduction effect decreases with increasing air gap for smaller fields (3 × 3 cm^2^), but for 5× 5 cm^2^ fields, it first increases and then decreases, or vice versa. This trend continues at a 30 cm pullback, where the reduction effect for 3× 3 cm^2^ fields diminishes with increasing air gap. These patterns result from the combined influences of pullback distance, air gap, and field size, with pullback having the greatest impact. A larger pullback has a large MCS between protons and RS, under a small air gap between the aperture and the phantom, enhancing its penumbra reduction. Penumbra size and reduction variations introduced by field size difference stem from differences in beam divergence. These factors collectively influence the penumbra and aperture effects in a non‐proportional manner, leading to the distinct patterns shown in Figure 3. Furthermore, these penumbra reduction patterns partially resemble those observed in single‐energy PBS proton irradiation with static and multi‐energy PBS IMPT with dynamic apertures.^39^, ^41^, ^43^, ^44^, ^55^

Beam‐specific brass apertures are typically designed to match the largest beam's eye view of the tumor cross‐section, making them most effective at the depth of the maximum tumor cross‐section. At other depths, the penumbra reduction effect is less effective due to the boundaries of the target projection being distant from those of the maximum projection cross‐section. To address these challenges more comprehensively in IMPT, some researchers have explored using dynamics ^39^, ^41^, ^56^ or spot apertures ^57^ to correct lateral penumbra at all depths for better reduction. However, the ultra‐high dose rate requirement of FLASH limits the application of these methods in SEBP FLASH technology.

In this study, the aperture was positioned immediately downstream of the URS and RCs. However, adjusting the distance between the URS and RCs and the aperture theoretically could optimize lateral penumbra under various conditions, providing additional tunable parameters for BP FLASH applications. Additionally, the thickness and margin size of the aperture are critical for effective BP FLASH implementation. Insufficient margins or excessive thickness may compromise dose coverage and significantly reduce the dose rate, while excessive margins or insufficient thickness can negate the benefits of the aperture.^37^ However, optimal parameter values require case‐by‐case analysis.

PBS delivery offers various methods for dose rate calculation. However, the biological mechanisms behind the FLASH effect remain unclear, and no consensus exists on the definition of the optimal dose rate for evaluating FLASH coverage. In our study, we used the ADR, a conservative approach. Based on this, we introduced V_40 Gy(RBE)/s_ to quantify FLASH dose rate coverage in OARs, applying multiple dose thresholds (0, 2, and 5 Gy(RBE)) across the full plan dose scenarios.^12^, ^58^, ^59^

Showcase simulations reveal that aperture use enhances GTV dose while significantly reducing OAR dose (Figure 5). However, this improvement comes with a notable decrease in V_40Gy(RBE)/s_ ratio for both GTV and brain for low‐dose region. Although some loss of FLASH‐related protection in the low‐dose region may occur, this is less concerning clinically. In contrast, the FLASH ratio, as measured by V_40Gy(RBE)/s_, remained well maintained in the high‐dose region with aperture use. Therefore, apertures may provide an overall dosimetric benefit by reducing lateral penumbra and dose spillage to OARs, thereby improving target dose conformity in range shifter‐based SEBP FLASH‐RT.

Our study employed brass apertures, contrasting with tungsten or nickel reports.^40^, ^55^ While tungsten offers slight dosimetric advantages over brass due to its higher density, the primary distinction lies in neutron production capacity. Although nickel has a lower neutron cross‐section, its higher processing difficulty and greater cost make it less economical compared to brass. Brass, in contrast, generates fewer neutrons upon proton impact than heavy metals (tungsten) and provides easier processing and better cost‐effectiveness.

## 5 CONCLUSION

This study demonstrates that applying a brass aperture to SEBP FLASH‐RT effectively reduces penumbra and OAR doses, particularly with large air gaps and range pullbacks. However, aperture usage diminishes ultra‐high dose rate coverage for OARs, potentially impacting FLASH‐sparing evaluation. Future research should focus on enhancing ultra‐high dose rate coverage in BP FLASH with aperture while preserving penumbra reduction for improved plan conformity. A 5 cm thick brass aperture with a 5 mm margin proved suitable for the study's irradiation conditions.

## AUTHOR CONTRIBUTIONS

Minglei Kang, Xuanqin Mou, Xueqing Yan, and Charles B. Simone II conceived and designed the study. Yangguang Ma, Balaji Selvaraj, Xingyi Zhao, and Chingyun Cheng collected the data. Yangguang Ma, Chin‐Cheng Chen, Longfei Diao, Yufei Wang, and Zhengda Wang performed the data analysis and interpretation. Yangguang Ma, Balaji Selvaraj, Yuntong Pei and Lele Liu developed the methodology and computational models. Yangguang Ma, Xingyi Zhao, Benjamin Durkee, Haibo Lin contributed to the software and validation. Yangguang Ma and Xingyi Zhao drafted the manuscript. All authors reviewed, edited, and approved the final version of the manuscript.

## FUNDING INFORMATION

The authors have nothing to report.

## CONFLICT OF INTEREST STATEMENT

The authors declare that they have no competing interests or personal relationships that could have appeared to influence the work reported in this paper.

## ETHICAL APPROVAL

The Ethics Committee of the authors’ institution reviewed the study protocol and determined that it was exempt from formal ethical approval.

## Data Availability

Research data are not shared.
